# The Association Between Emotional Intelligence and Suicidal Behavior: A Systematic Review

**DOI:** 10.3389/fpsyg.2018.02380

**Published:** 2018-11-30

**Authors:** Elena Domínguez-García, Pablo Fernández-Berrocal

**Affiliations:** Department of Basic Psychology, Faculty of Psychology, University of Málaga, Málaga, Spain

**Keywords:** emotional intelligence, suicide, suicidal behavior, suicide risk, emotional skills, emotional competence, systematic review

## Abstract

**Background:** Some meta-analyses have demonstrated the association between emotional intelligence (EI) and different health indicators. With the increase of suicide cases in the world, more and more professionals have been interested in the link between both variables.

**Aim:** To study all the available evidence on the association between EI and suicidal behavior.

**Method:** We systematically reviewed all available literature (in English or Spanish) on the relationship between both variables through the main databases.

**Results:** Twenty-five articles were included. EI and suicidal behavior correlated inversely in almost all the articles that the Emotional Quotient Inventory (EQ-i), The Emotional Intelligence Test (EIT), The Spanish Wong and Law Emotional Intelligence Scale (WLEIS), and The Schutte Emotional Intelligence Scale (EIS/SSEIT), Barchard's Emotional Intelligence Scale, were used, that is, the higher suicidal behavior level the lower the EI score. The same results were found in two out of four investigations that used the Trait Meta-Mood Scale (TMMS-24) between clarity (emotional understanding) and emotional repair (emotional regulation) and suicidal behavior. Two out of three studies that used the Mayer-Salovey-Caruso Emotional Intelligence Test (MSCEIT) found that only the Strategic component of EI (emotional understanding and regulation) was a protective factor.

**Conclusions:** The results appear to indicate that a high level of EI plays an important role in protecting against suicidal behavior, and should thus be integrated into suicide prevention programs.

## Introduction

The World Health Organization (WHO) estimated that 804,000 people died from suicide in 2012 (11.4 per 100,000 people), with these numbers being higher in men than women. Over the past 45 years, the suicide rate has risen by 60% and has been established as the second cause of death among the 15–29 year age category, with 50 non-fatal suicide attempts for each suicide. Risk factors linked to interpersonal relationships and the community include war and disaster, discrimination, abuse, violence and conflicts, perception of isolation, and stresses of acculturation (displaced people or indigenous persons), whilst risks at the individual level include chronic pain, mental disorders, poor financial situation, family history of suicide, previous non-fatal suicide attempts, and the harmful abuse of alcohol. Prevention strategies should be focused on encouraging protective factors such as positive coping strategies, a strong personal belief, and strong interpersonal relationships (WHO, 2014).

Arsenault-Lapierre et al. (2004) carried out a meta-analysis on the psychiatric diagnosis in 3,275 suicides, in which the results revealed that 87.3% of people who committed suicide had been diagnosed with a mental disorder. In addition, two of the most common mental disorders among people who committed suicide were affective disorders (present in 43.2% of suicide cases) and drug abuse disorders (present in 25.7% of suicide cases), comorbidity being present in most cases.

Law and Tucker (2017) studied how repetitive negative thinking affects the risk of suicide. Rumination—a relevant symptom present in affective disorders—occurs due to the perception of a discrepancy between the current state of a person and the goals to be achieved (Watkins and Nolen-Hoeksema, 2014). It has been found that the perception of one's inability to use emotional regulation strategies (Miranda et al., 2013) and change strategies in a given situation, predicts suicidal ideation in 2 or 3 years through brooding and rumination, initiating or maintaining a sequence from rumination to hopelessness and thoughts of suicide. In addition, hopelessness, perception of entrapment (Teismann and Forkmann, 2017) and lack of optimism (Tucker et al., 2013) strengthen the relationship between suicide ideation and rumination. The association between the aforementioned variables may be intensified by the impulsive use of provocative and painful behaviors when coping with negative emotions caused by rumination. Furthermore, suicidal behaviors have been found to correlate with depressive symptoms (Selby et al., 2007; Chu et al., 2016).

Another relevant point regarding affective disorders is the association between early maladaptive schemas of emotional deprivation, social isolation, shame and abandonment, and history of suicide attempts. Ahmadpanah et al. (2017) showed that, unlike the control group, people with major depressive disorder scored higher on the aforementioned variables. Those with maladaptive schemas do not expect others to support or take care of them, since they feel emotionally deprived, as if they did not perceive enough affection, warmth, and attention. Normally these people do not express their emotions, since they do not expect any kind of emotional support. Depression can occur as a result and, depending on its durability and severity, the risk of suicide increases.

With respect to the second cause of suicide reported by Arsenault-Lapierre et al. (2004), suicide among young people has increased dramatically in recent decades (Peters et al., 1998), and in young people this increase has been associated with an increase in the consumption of drugs and alcohol (Brent, 1995; Ilomaki et al., 2007; West et al., 2010).

The systematic review by Pompili et al. (2012) studied the relationship between substance abuse and suicidal risk among adolescents, showing that regular drug abuse (cannabis, alcohol, heroine, cocaine) increases depression, hopelessness, and disruptive and suicidal behavior.

### Definition of suicidal behavior

The WHO defines suicide as “an act with a fatal outcome which the deceased, knowing or expecting a fatal consequence, had initiated and carried out with the purpose of provoking the changes that he desired” (WHO, 1986), whilst considering suicidal behavior as “a range of behaviors that include thinking about suicide (or ideation), planning for suicide, attempting suicide and suicide itself” (WHO, 2014). For the purposes of our systematic review, we will adopt these definitions.

### Emotional intelligence (EI)

The theoretical models of EI can be categorized into two branches (Mayer et al., 2008): EI skill models (mental abilities) and EI mixed models (capacities, traits, and abilities). The first considers EI as a set of abilities that are part of the cognitive processes, and which constitute a form of intelligence that fosters the ability to “accurately perceive, value and express emotions, the ability to access and/or generate feelings that facilitate thinking, to understand emotions and to reason emotionally, and finally the ability to regulate one's own and others' emotions” (Mayer and Salovey, 1997). Ability EI is mainly assessed using performance tasks. The mixed model considers EI as a set of personality traits that determines the tendency of a person to manage their emotions in a certain way (Goleman, 1995; Bar-On, 1997; Petrides et al., 2007). This theoretical approach mainly uses self-reported questionnaires.

### EI and suicide

According to Fernández-Berrocal and Extremera (2016), there is an increasing interest in the link between emotions and health, prompting authors to conduct meta-analyses on the relationship between mental and physical health and emotional skills (Schutte et al., 2007; Martins et al., 2010). The results reveal a strong correlation between self-report ability EI and self-report mixed EI and health indicators. However, a moderate correlation has been found between health and performance-based ability EI scores.

It has been demonstrated that a stable and moderate deficit in the ability to decode mental states and emotional stimuli can be caused by difficulties in social interaction in people with Major Depressive Disorder (Hall et al., 2009; Kohler et al., 2011; Manstead et al., 2013; Weightman et al., 2014). In addition, evidence shows that a low EI score correlates negatively with depression, and thus contributes toward reducing the ability to understand and manage emotions, skills associated with the prefrontal cortex (Hertel et al., 2009; Kwako et al., 2011; Sawaya et al., 2015). Moreover, a study by Sawaya et al. (2015) found fewer functional connections between regions involved in emotional regulation and the anteromedial region in people with Major Depressive Disorder. They also found a significant correlation between MSCEIT scores and functional connectivity in the ventromedial prefrontal cortex. The results indicate that people with Major Depressive Disorder struggle to perceive and manage emotions and to participate in positive social interactions.

In contrast, Zeidner et al. (2012) have argued that emotional skills play a crucial role in promoting positive emotions and well-being. In particular, people with high EI are more likely to create and maintain close relationships, improving their subjective well-being (Lopes et al., 2005) whilst using more effective coping strategies such as expressing emotions and feelings, rather than maladaptive strategies such as avoidance or rumination (Matthews et al., 2006). Moreover, they tend to experience less emotional distress when facing a stressful situation, increasing positive affect (Gohm et al., 2005) and are more able to maintain higher self-esteem and self-efficacy, mitigating the influence of negative events (Salguero et al., 2015). This has been confirmed by several meta-analyses reporting that performance-based measures of EI skills are associated with subjective well-being (Sánchez-Álvarez et al., 2016). Furthermore, life satisfaction may correlate positively with EI, as revealed in a study carried out by Mayer et al. (1999) in which a weak to moderate correlation was found between life satisfaction and EI measured by the Multifactor Emotional Intelligence Scale. More recent studies have provided support for these positive associations between EI and current life satisfaction or retrospective life satisfaction (Brackett et al., 2006; MacCann and Roberts, 2008; Extremera et al., 2011; MacCann et al., 2016).

Gallagher and Miller (2018) carried out a systematic review of the literature on suicidal thoughts and behavior in children and adolescents, concluding that, in spite of the low number of articles that link EI with suicidal behavior, the literature suggests the abilities of adolescents to understand and cope with self-emotions reduce suicidal risk and promote resilience. Furthermore, a study by Rivers et al. (2013) examined how emotion skills could be a protective factor for risky behaviors among college students, and found that there was a negative correlation between EI and risky behaviors such as substance abuse, among other relevant variables.

These results are of clear relevance, given that EI could act as a protective factor for the general population, particularly for adolescents, since depression is the first cause of suicide in our society, followed by substance abuse.

The aim of this article is to carry out, as completely as possible, a systematic review of all the existing Spanish and English literature to date on the relationship between EI and suicidal behavior. On the basis of this review, the intention is to present a perspective of the current research situation in this field, as well as offering proposals for future lines of research that could overcome the limitations found in the current literature.

## Methods

### Literature search procedures

A consultation was carried out through the following databases: Web of Science, Scopus, Medline, Pubmed, PsycINFO, ProQuest, Riuma, Dialnet, and Google Scholar. A first search was conducted between 17 April 2017 and 21 April 2017 without any limitations on the year of publication of the articles. Firstly, we conducted a superficial search of articles about EI and suicide (without the need for both factors to be related), in order to identify the keywords in the search itself. Then, we established the following terms to define the search criteria: “emotional intelligence” or “emotional competence,” and one of the following keywords: “suicide,” “suicidal behavior,” “suicidal ideation,” “suicidal attempt,” both in English and Spanish. After obtaining the results from the various databases, we proceeded to read all of the titles and abstracts, selecting the possible candidates for our review and eliminating duplicate studies. Finally, an in-depth reading of each of the articles was carried out. The search was completed by a reviewer and supervised by an expert.

**Figure 1:**
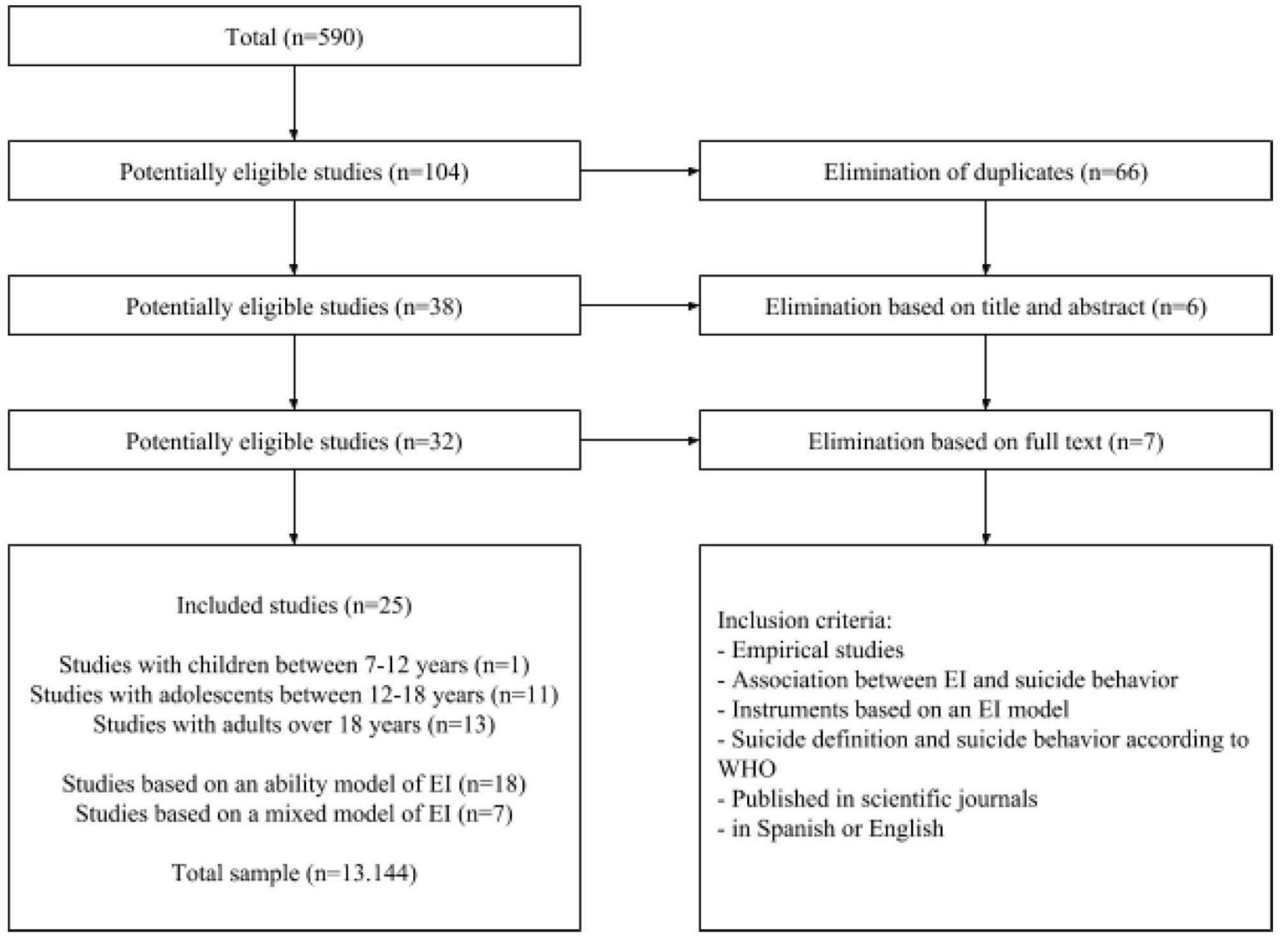
Flowchart of study selection.

A new updated search was conducted in October 2018 to find out if new studies had been published. In this case, we established as a time limitation the studies that has been published between 2017 and 2018. The flowchart of the study selection can be seen in Figure 1.

### Criteria for selecting studies

The following inclusion criteria were adopted: the study must be empirical, and based on the relationship between EI and suicide; the work should adopt a definition of suicidal behavior similar to the one given by WHO; the study must employ EI instruments based on theoretical models; the work should be published in scientific journals; and in Spanish or English.

### Materials and instruments used in articles

#### EI assessment

The following are the characteristics of the instruments and tasks that have been used in the different studies, following a classification system based on the theoretical model adopted and the different measures used. Thus, the studies involved either performance or self-report tasks based on the ability model of EI, or self-report instruments based on EI mixed models (Joseph and and Newman, 2010). The psychometric properties are indicated if provided by the study.

##### Performance-based instruments based on ability models

The Mayer-Salovey-Caruso Emotional Intelligence Test (MSCEIT; Mayer et al., 2003) consists of 141 items that measure the ability to solve emotional problems corresponding to the four-branch model of EI developed by Mayer and Salovey (1997) scored in two areas: experiential EI (emotional perception and facilitation) and strategic EI (emotional understanding and regulation). The test has a high internal consistency (α = 0.93), good test–retest reliability and excellent construct and current validity. Some studies have employed their version of this test for young people, the Mayer-Salovey-Caruso Emotional Intelligence Test: Youth Version, Research Version 1.0. (MSCEIT: YV-R; Mayer et al., 2005).

##### Self-report instruments based on ability models

The Schutte Emotional Intelligence Scale (EIS/SSEIT, Schutte et al., 1998) consists of 33 items categorized into three areas: emotional perception and expression, emotional regulation, and the use of emotions in problem solving. It has internal consistency from 0.87 to 0.90, a test–retest reliability of 0.78 and evidence of construct validity. Some authors have used a Chinese adaptation, the Chinese Emotional Intelligence Scale (C-EIS-R; Chan, 2003).

The Objective emotion perception test consisted of six short stories, each one followed by seven emotions and a related 7-adjective mood scale (Mayer and Geher, 1996).

The Spanish adaptation of the Trait Meta-Mood Scale (TMMS-24; Fernández-Berrocal et al., 2004) evaluates the three categories of EI—attention to emotion, clarity, and emotional repair—through 24 items, with an internal consistency of 0.90, 0.90, and 0.86, respectively, and a test–retest reliability of 0.60, 0.70, and 0.83, respectively.

The Spanish Wong and Law Emotional Intelligence Scale (WLEIS; Wong and Law, 2002) evaluates four dimensions: self-emotion appraisal, other-emotion appraisal, use of emotion, and regulation of emotion. The test has been proven to have good psychometric properties in Spanish populations.

Emotional Intelligence scales (Barchard, 2001) assess EI through 68 items. In one article it was translated into Arabic (Madbouly et al., 2017). Reliability was 0.67.

##### Self-report instruments based on mixed models

The Emotional Quotient Inventory (EQ-i; Bar-On, 1997) consists of 133 items contained in 15 subscales and 5 major factors: interpersonal, intrapersonal, stress management, adaptation, and general mood. The EQ-i has very good reliability for internal consistency. Some research studies have employed the youth version, the EQ-i: YV, Bar-On Emotional Quotient Inventory: Youth Version (Bar-On and Parker, 2004), which has been shown to have internal consistency across scales ranges from *r* = 0.67 to 0.87 for males and *r* = 0.65 to 0.87 for females. Test–retest reliability ranges from *r* = 0.77 to 0.88.

In one study (Mamani et al., 2018) the Peruvian adapted version of EQ-I (BarOn-ICE; Ugarriza Chávez and Pajares del Águila, 2005) was used. Cronbach value of the test was 0.77.

The Emotional Intelligence Test (EIT; Chadha, 2001) is based on Goleman's model (1995) and assesses emotional sensitivity, maturity, and competence.

#### Suicidal behavior assessment

The instruments used to assess suicidal behavior do not follow a specific model. The psychometric properties are indicated if provided by the study.

##### Reynolds scales

The Adult Suicidal Ideation Questionnaire (ASIQ; Reynolds, 1987) is a 25-item self-report measure that evaluates suicidal ideation.

The Suicidal Ideation Questionnaire (SIQ; Reynolds, 1991) assesses the frequency of suicidal ideation through 33 items. Several studies showed that this test has good internal consistency (α = 0.94) and adequate construct validity (Pinto et al., 1997).

##### Beck scales

The Beck Scale for Suicide Ideation (BSSI; Beck et al., 1979) evaluates the intensity of suicidal ideation through 19 items and four subscales.

The Beck Depression Inventory (BDI-II: Beck et al., 1996) is a 21-item scale questioning whether suicidal behavior is being experienced.

##### Plutchik suicide risk scale (SRS; plutchik et al., 1989)

This is a self-report questionnaire that evaluates the risk of suicide through 15 items adopting a dichotomous response format. This scale presents good reliability and validity.

##### Self-injurious thought and behaviors interview (SITBI; nock et al., 2007)

This is a short structured interview that assesses the presence, frequency, and other characteristics of the range of self-injurious thoughts and behaviors, including suicidal ideation or non-fatal suicide attempts. This test presents good inter-rater and test–retest reliability and concurrent validity.

##### Suicidal tendency subscale of the millon adolescent clinical inventory (MACI; millon, 2004)

This questionnaire assesses personality features, clinical syndromes and concerns in adolescents using seven scales, including a suicidal tendency scale (25 items).

##### The child-adolescent suicidal potential index (CASPI; pfeffer et al., 2000)

This test evaluates the risk of suicide through 30 items categorized into three subscales: anxiety-impulsive depression, suicidal ideation, or non-fatal suicide attempts and family distress. The internal consistency is 0.90 and the test–retest reliability is 0.76.

##### Suicidal ideation subscale of the general health questionnaire (chan, 2005)

This is a 4-item subscale that uses a Likert-type response scale. Some of the items are: “I feel that life does not deserve to be lived” or “I have thoughts of ending my life.”

##### Suicidal ideation subscale of the suicidal risk scale (C-SIS; tse and bagley, 2002)

This test is composed of 13 items that evaluate the intensity of suicidal ideation. This scale has been shown to have high internal consistency (α = 0.92), split-half consistency (α = 0.88), and test–retest reliability (*r* = 0.72).

##### Suicidal behaviors questionnaire—revised (SBQ-R; osman et al., 2001)

The SBQ-R consists of four items assessing lifetime suicidal ideation and non-fatal attempts, frequency of suicidal ideation over the past year, threat of suicidal behavior, and self-reported likelihood of future suicidal behavior. It has been shown to have good reliability and validity when used with both college students and clinical samples.

##### MINI international neuropsychiatric interview (sheehan et al., 1998)

In the study by Kopera et al. (2018) they assessed lifetime history of suicide attempts with a question from the MINI International Neuropsychiatric Interview: “During your lifetime, have you ever tried to commit suicide?”

## Results

### EI theories and assessment methods

As mentioned previously, there are several explanatory models of EI (ability or mixed) that use their own evaluation instruments, which usually consist of performance or self-report tasks depending on what the theoretical model considers to be most appropriate. In our case, of the 25 selected articles, 18 are based on the ability model of Mayer and Salovey (MSCEIT, MSCEIT: YV-R, TMMS-24, Barchard Emotional Intelligence Scale, WLEIS, EIS/SSEIT, and C-EIS-R), six on the mixed model of Bar-On (EQ-i), and one on the mixed model of Goleman (EIT).

### General characteristics of the included studies

The most relevant information from the sample in general terms and from the studies included in this systematic review can be viewed in Table 1 (sample in general terms), Table 2 (studies with children), Table 3 (studies with adolescents), and Table 4 (studies with adults), respectively. In order to present the data in a more appropriate way, we divided the studies by age (children, adolescents, or adults), and grouped the results according to the instrument applied in each case.

**Table 1:** Sample in general terms.

	**Males**	**Females**	**Sex not specified**	**Total**
Children (< 12 years)	87 (67.44%)	42 (32.56%)	–	129
Adolescents (12–18 years)	4.591 (48.8%)	4.450 (47.3%)	367 (3.9%)	9.408
Adults (>18 years)	1.375 (38.12%)	2.173 (60.24%)	59 (1.64%)	3.607
Total	6.053 (46.05%)	6.665 (50.71%)	426 (3.24%)	13.144

**Table 2:** Selected studies on EI and suicidal behavior in children (7–12 years old).

**Study**	**Type of suicidal behavior**	**EI instruments**	**Sample**	**Main results**
Bodzy et al., 2016	SI and NSA	EQ-i:YV-S	129 children (42 females)	The SI group scored higher in the EQ-i than in the NSA group

*SI, Suicidal ideation; NSA, Non-fatal suicide attempt; EQ-i: YV, Bar On Emotional Quotient Inventory: Youth Version (Bar-On and Parker, 2004)*.

**Table 3:** Selected studies on EI and suicidal behavior in adolescents (12–18 years old).

**Study**	**Type of suicidal behavior**	**EI instruments**	**Sample**	**Main results**
Abdollahi et al., 2016	SI	EIS	202 adolescents (105 females)	High levels of EI correlate with low SI and low perceived stress. EI moderates both variables
Abdollahi and Talib, 2015	SI	EIS	202 adolescents (105 females) Same sample of the previous study	EI moderates the link between brooding, reflective styles and SI. EI correlates negatively with SI in non-attempters
Kwok et al., 2015	SI	C-EIS-R	127 adolescents (251 females)	No association between EI and SI was found
Kwok and Shek, 2010	SI	C-EIS-R	5.557 adolescents students (46,9% females)	There is a negative correlation between SI and EI. EI negatively predicts SI
Extremera et al., 2018	SI	WLEIS	1660 adolescents (50.4% females)	Lower EI scores are associated with a higher suicidal risk in cyberbullyism victims
Limonero et al., 2018	SI	TMMS-24	144 young university students (105 females)	Low levels of clarity and emotional regulation correlates with a higher suicidal risk
Alizadeh and Aveh, 2016	SI	EQ-i	367 adolescent students	EI predicts and correlates negatively with SI
Mamani et al., 2018	SI	EQ-I (BarOn ICE)	33 females with SI	Intervention improved suicide ideation and EI in the components of intrapersonal, stress management and general mood scale
Zavala and López, 2012	SI	EQ-i: YV	829 adolescent students (435 females)	High EI correlates inversely with depression and suicide
Madbouly et al., 2017	NSA	Barchard's Emotional Intelligence Scale	36 adolescents with SA (89% females)	Intervention improved suicide risk and EI, but there was no significant correlation between these variables
Cha and Nock, 2009	SI and NSA	MSCEIT:YV-R	54 adolescents (46 females)	Strategic EI is a protective factor for suicidal behavior

*SI, Suicidal ideation; NSA, Non-fatal suicide attempt; EIS, Schutte Emotional Intelligence Scale (Schutte et al., 1998); C-EIS-R, Chinese Emotional intelligence Scale (Chan, 2003); EQ-i, The Emotional Quotient Inventory (Bar-On, 1997); TMMS-24, The Spanish adaptation of Trait Meta Mood Scale (Fernández-Berrocal et al., 2004); EQ-i:YV, Bar-On Emotional Quotient Inventory: Youth Version (Bar-On and Parker, 2004); MSCEIT: YV-R, Mayer-Salovey-Caruso Emotional Intelligence Test: Youth Version, Research Version 1.0 (Mayer et al., 2005)*.

**Table 4:** Selected studies on EI and suicidal behavior in adults (over 18 years).

**Study**	**Type of suicidal behavior**	**EI instrument**	**Sample**	**Main results**
Aradilla-Herrero et al., 2014	SI	TMMS-24	92 nursery students (75 females)	Emotional attention predicts SISI correlates negatively with clarity and repair
Caballero et al., 2015	SI	TMMS-24	44 college students (32 females)	Low SI correlated with better understanding and regulation of negative emotions. High SI correlated with a higher emotional attention
Ceballos and Suárez, 2012	SI	TMMS-24	157 college students (106 females)	EI and SI correlate negatively
Extremera and Rey, 2016	SI	WLEIS	1.125 unemployed adults (619 women)	EI moderates the relationship between happiness and life satisfaction and SI. Low EI correlates with higher SI
Mérida-López et al., 2018	SI	WLEIS	Study 1: 438 students (270 females) Study 2: 330 students (264 females)	EI correlated negatively with suicidal thoughts and behaviors in both studies and EI may act as a protective factor against suicidal risk
Ciarrochi et al., 2002	SI	EIS Objective emotion perception test	302 college students (232 females)	High emotional perception correlates positively with SI, as opposed to a high management of other's emotion and SI
Kopera et al., 2018	SI and NSA	SSEIT	80 inpatients entering an alcohol treatment program (56 females)	Mood regulation fully mediated the association between depression and history of suicide attempts. No correlation was found between this latter variable and other EI components (emotional perception, understanding and utilization of emotions)
Kwok, 2014	SI	C-EIS-R	302 college students (228 females)	EI negatively correlates and predicts SI
Moayedi et al., 2014	NSA	EQ-i	100 adults (50 females with SA)	NSA group scored significantly lower in EI, as opposed to the control group
Rahgozar et al., 2011	NSA	EQ-i	60 adults (30 with SA)	NSA group scored significantly lower in EI, as opposed to the control group
Hazra and Dasgupta, 2011	SI	EIT	200 adult students (100 females)	EI negatively correlates with SI
Karim and Shah, 2014	SI	MSCEIT	192 college students (91 females)	Strategic component of EI predicts SI
Paradiso et al., 2016	SI	MSCEIT	185 war veteran males (46 with SI)	Lower EI levels are associated with greater SI

*SI, Suicidal ideation; NSA, Non-fatal suicide attempt; TMMS-24, The Spanish adaptation of Trait Meta Mood Scale (Fernández-Berrocal et al., 2004); WLEIS, The Spanish Wong and Law Emotional Intelligence Scale (Wong and Law, 2002); EIS/SSEIT, The Schutte Emotional Intelligence Scale (Schutte et al., 1998); C-EIS-R, Chinese Emotional intelligence Scale (Chan, 2003); EQ-i, The Emotional Quotient Inventory (Bar-On, 1997); EIT, Emotional Intelligence Test (Chadha, 2001); MSCEIT, Mayer-Salovey-Caruso Emotional Intelligence Test (Mayer et al., 2003)*.

### Results of the selected studies according to age range

#### Studies with children (under 12 years old)

The study by Bodzy et al. (2016) compared a sample of hospitalized children in psychiatric settings, dividing them between those with suicidal ideation and those who had a non-fatal suicide attempt according to their hospital reports and the CASPI questionnaire, whilst their EI levels were compared by using the EQ-i: YV-S. The total score on the EQ-i: YV-S was higher in the group with only suicidal ideation than in the subsample of children with a reported history of non-fatal suicide attempts, although no subscale was an independent predictor of suicide. The main data can be viewed in Table 2.

#### Studies with adolescents (12–18 years old)

##### Studies using self-reported EI tests

The investigations of Kwok and Shek (2010), Abdollahi and Talib (2015), and Abdollahi et al. (2016) used EIS to analyze the relationship between EI and suicidal ideation, among other variables. All the studies found a negative correlation between both variables, with EI being a protective factor. The first article explored the role of EI in the relationship between brooding and reflection and suicidal ideation (measured by the SIQ) among Iranian hospitalized adolescents. The results showed that a high level of both variables and a low level of EI correlates with a higher suicidal ideation, with EI being a mediator between rumination and suicidal ideation. In a later article using the same results for the same sample, (Abdollahi et al., 2016) found that 39% of the variance in suicidal ideation was explained by EI and perceived stress among the depressed adolescents. Lower suicidal ideation and lower perceived stress was observed in non-depressed patients with high levels of EI, as opposed to those with low levels of EI, indicating that EI also moderated the relationship between suicidal ideation and perceived stress. Kwok and Shek (2010) found an inverse correlation between suicidal ideation (measured by C-SIS) and emotional self-regulation, social skills, and use of emotion, as measured by subscales of the Chinese version of EIS (C-EIS-R, Chan, 2003) in adolescents from 42 schools in Hong Kong, the New Territories, and Kowloon. In contrast, in a later study by Kwok et al. (2015), using the same instruments, suicidal ideation was not found to be significantly associated with EI. However, empathy was found to moderate the relationship between physical abuse and suicidal ideation, but only in females.

Extremera et al. (2018) explored the relationship between EI, suicidal risk, self-esteem, and cyberbullying victimization in adolescents and examined whether EI could play a role as a moderator of the relationship between those variables. They used WLEIS to assess EI and SBQ-R to evaluate suicide risk, along with other questionnaires to test the other variables. The results revealed that adolescents with greater EI were less likely to show symptoms of low self-esteem and suicidal ideation in comparison with those whose EI scores were lower.

Madbouly et al. (2017) carried out a pre–post intervention with the aim of exploring the effects of an intervention on suicide ideation and EI. The questionnaires used were the Barchard's Emotional Intelligence Scale and Beck's Suicidal Ideation Scale. The intervention improved both variables, but there was no significant correlation between suicide ideation and EI.

The study by Limonero et al. (2018) analyzed the relationship between perceived EI (by using the TMMS-24) and negative affect, life satisfaction, and suicide risk in adolescents (by using the SRS). The results showed that suicidal risk correlated negatively with clarity and emotional regulation and life satisfaction, emotional regulation being a predictor of suicidal risk.

Zavala and López (2012) and Alizadeh and Aveh (2016), who used the EQ-i to measure EI, found similar results, concluding that there was a negative correlation between EI and suicidal tendency, with EI being a predictor of suicidal tendency. While in the first study the relationship between EI and suicidal tendency among adolescents in Gachsaran (Iran) was measured by using the BSSI, the second investigation evaluated, through the MACI, certain psychosocial risk factors (including suicidal tendency), and their relationship with perceived EI through the EQ-i: YV in adolescents from León (Mexico). Zavala and López (2012) found that only the interpersonal component of EI correlated negatively with suicidal tendency. The remaining scales of the EQ-i: YV showed no significant correlation with autolytic behavior.

Mamani et al. (2018) carried out a test–retest design study in which they evaluated the effect of an intervention on emotional intelligence and suicidal risk by using the BSSI and the Peruvian adaptation of EQ-i. The results indicate a post-intervention improvement of emotional intelligence levels and a decrease in suicidal risk.

##### Studies using performance EI tests

Cha and Nock (2009) analyzed, among other aspects, the relationship between EI (measured by MSCEIT: YV-R) and suicidal behavior (measured by SITBI) in adolescents from various nationalities that had been sexually abused in their childhood, both with and without a history of non-fatal suicide attempts. The results revealed that EI moderated the relationship between the history of sexual abuse and suicide behavior in the past, finding a strong relationship between suicidal tendency in participants with low IE. Specifically, the Strategic area of EI (understanding and emotional regulation) appeared to be a protective factor against suicide behavior.

The main data can be viewed in Table 3.

#### Studies with adults (over 18 years old)

##### Studies using self-reported EI tests

Aradilla-Herrero et al. (2014) assessed the relationship between suicide risk and EI among a sample of nursing students by using the SRS and the Spanish version of TMMS-24, respectively. The linear regression analysis revealed the predictive role of emotional attention in suicidal risk, whereas suicidal behavior showed a significant negative correlation with clarity and emotional repair. A study by Ceballos and Suárez (2012) with Psychology university students from Colombia again evaluated EI with the same instrument, and suicidal ideation through the BSSI, finding a negative correlation between emotional repair and suicidal ideation. However, no significant association was found with the other variables. As in the previous studies, the investigation by Caballero et al. (2015) revealed similar results in other students from the same university, showing that the greater the emotional attention, the greater the risk of suicide, while the improvement in clarity and emotional repair was associated with a lower suicidal tendency.

In a sample of unemployed Spanish adults, Extremera and Rey (2016) evaluated the association between EI (WLEIS) and suicidal ideation (SBQ-R) along with other variables, finding that lower scores in perceived EI increased the likelihood of suicidal behaviors, whilst perceived EI additionally moderated the relationship between suicidal ideation and life satisfaction and happiness. The same authors conducted two new independent studies (Mérida-López et al., 2018) with the same instruments. In the first study, they evaluated the associations between self-report EI, suicide risk and psychological distress, with the expectation that this last variable would operate as a mediator of the relationship with suicide risk. In the second study, they prospectively tested the proposed model in a sample of college students, assessing the effects of self-report EI on suicide risk over a 2-month period. The results revealed that EI correlated negatively with suicidal thoughts and behaviors in both studies and that EI may act as a protective factor against suicidal risk.

EIS was used as a measure of EI in two investigations. Ciarrochi et al. (2002), assessed the relationship between EI (through EIS and the Objective emotion perception test) and suicidal ideation through ASIQ. The analysis of the data showed that a greater emotional perception was associated with higher levels of depression and suicidal ideation in stressful situations. However, participants who scored high on regulating the emotions of others responded to stress with a lower level of suicidal ideation and depression. Kwok (2014) assessed the relationship between EI (C-EIS-R) and suicidal ideation (C-SIS), finding that all components of EI, but not empathy, correlated inversely with suicidal ideation, with EI being a predictor of this variable.

Kopera et al. (2018) explored the association between self-reported EI (by using the SSEIT) and prevalence of suicide attempts (by using a question from the MINI International Neuropsychiatric Interview). It was found that individuals with lower self-reported emotional regulation were more likely to show a higher prevalence of attempted suicide. In contrast, emotional perception, understanding, and utilization of emotions were not related to suicidal attempts. However, emotion regulation was found to mediate the association between depression and history of suicide attempts, and the association between neuroticism and suicide attempts.

Rahgozar et al. (2011) and Moayedi et al. (2014) used the EQ-i as a measure of EI and obtained the same results. In both studies, groups with a history of non-fatal suicide attempts scored significantly lower on EI than the control group, with the latter group scoring higher on all subscales. Similar results were also found by Hazra and Dasgupta (2011). They evaluated the relationship between EI (EIT) and suicidal ideation (ASIQ) among students from Kolkata (India), finding an inverse correlation between both variables.

##### Studies using performance EI tests

Karim and Shah (2014) and Paradiso et al. (2016) used MSCEIT to evaluate the relationship between EI and suicidal ideation, finding in both investigations a negative correlation between these constructs. In the first article, the results showed that the Strategic component of EI—and not the Experiential—was a significant predictor of suicidal ideation as measured through the General Health Questionnaire in students from Pakistan and France. In the second article, a negative correlation was found between most of the EI components (understanding, facilitation, and emotional regulation) and suicidal ideation (assessed by the BDI-II) in a sample of adult war veterans.

The main data can be viewed in Table 4.

## Discussion

In this systematic review, we have analyzed the existing literature on the relationship between EI and suicidal behavior, finding a total of 25 articles with relevant results. Although some meta-analyses have previously been conducted on the relationship between EI and different health indicators (Schutte et al., 2007 and Martins et al., 2010), to our knowledge, no systematic review or meta-analysis has yet been completed with the variables studied in the present research.

According to the results obtained, in almost all the articles that EQ-i, EIT, WLEIS, and EIS were used as a self-reported measure of EI, a significant inverse correlation was found between this variable and suicidal behavior, with EI also being a good predictor of suicidal behavior.

It is important to note that both Ciarrochi et al. (2002) and other (later) studies found that an extremely high level of emotional perception is positively associated with suicidal behavior, while a high level of emotion regulation of others correlates negatively with this construct.

With respect to the studies that used the TMMS-24 to evaluate EI, two out of four investigations obtained a positive correlation between emotional attention (perception and emotional identification) and suicidal behavior, which is in agreement with previous research. In contrast, in almost all the articles that used the same EI measurement, clarity (emotional understanding) and emotional repair (emotional regulation) are inversely related to suicidal behavior. Consistent with these results, two out of three studies found that, when using the MSCEIT, only the Strategic component of EI (emotional understanding and regulation) was a protective factor for suicidal behavior. However, in the last study (Paradiso et al., 2016), emotional facilitation (included in the Experiential component) was also added as a protective element.

Similar results were found across all age periods, even though the samples of participants were composed of different groups and countries.

The results strongly agree with those found in the literature on the association between suicide risk and EI, and the role of the latter as a protective factor. The reasons for why EI has a protective capacity could be linked to its negative correlation with depression and risky behaviors such as substance abuse; its role in promoting positive emotions and resilience; the creation and maintenance of close relationships; the use of effective coping strategies rather than maladaptive strategies; the experience of less emotional distress when coping with a stressful situation; the maintenance of higher self-esteem and self-efficacy that mitigates the influence of negative events; and the increase of subjective well-being and life satisfaction, as mentioned above.

Some limitations of this study are worth noting. First, analysis of the results was made difficult by the fact that each one adopted a different theoretical model and, consequently, different instruments. In addition, we do not know the effect size of the correlation between both variables, so it would be interesting to carry out a meta-analysis in the future. In some studies, certain socio-demographic data are not correctly specified, so the total number of people corresponding to each sex is unknown, and this may be a variable of interest when analyzing the data. It is important to point out that almost all the studies are quasi-experimental and transversal investigations and in only two cases was a test–retest design employed. It is thus more complicated to generalize the results to certain groups, whilst it is also challenging to analyze the persistence of results in the long-term. Finally, only two studies applied an intervention but, despite the post-test improvement in EI and suicide risk, there was no significant correlation between both variables.

Future research should focus on studying gender differences since there are variations in suicidal risk, with males showing a higher frequency of Serious Suicide Attempts (SSA) than females (Freeman et al., 2017). Furthermore, the literature shows that self-report ability EI and gender moderate the association between EI and Major Depressive Disorder (Fernández-Berrocal and Extremera, 2016), which could improve the quality of life in people with affective disorders, preventing the risk of suicide.

Even though WHO has proposed strategies and intervention guidelines that have proved useful (WHO, 2014), it is still necessary to design definitive programs that address all aspects related to suicide, depending on the age and the characteristics of the population. The goal of preventing suicidal behavior will be crucial in childhood and adolescence, a period in which it may be beneficial to introduce a number of interventions including EI training, paying attention to mental disorders and other associated risk factors, and enhancing protective factors through the development of programs that could increase their effectiveness such as “Signs of Suicide” (SOS; Schilling et al., 2016). Similarly, validating both prevention and intervention programs in adulthood could be an interesting approach for improving existing programs, such as the elderly suicide prevention programs (Lapierre et al., 2011).

Certain social and emotional-learning validated programs such as the SEL Training Intervention (Castillo-Gualda et al., 2017) or RULER (Nathanson et al., 2016) have significantly improved the emotional skills of both young students and adults. The former was a 3-year intervention, the aim of which was to improve the following EI skills: accurate perception, appraisal, and expression of emotions; awareness of feelings and ability to generate emotions to facilitate thought; understanding of emotions, regulation of emotions in order to promote emotional and intellectual growth. RULER is an EI program for students, which has shown to be effective in enhancing student outcomes and includes a set of practical exercises. Five skills are enhanced during the intervention: perception of emotions in the self and others, understanding emotions, labeling emotions with a diverse and accurate vocabulary, expressing emotions, and effectively regulating emotions. These programs could be used to reduce suicidal risk by improving EI skills.

In conclusion, according to Mayer and Salovey's theory (Salovey and Mayer, 1990; Mayer and Salovey, 1997; Mayer et al., 2016), EI is a mental ability, and thus it can be learned and improved by implementing intervention programs such as RULER. In addition, the literature reports that, regardless of age, nationality, or EI instruments, it has been found that the EI variable plays an important role as a protective factor for suicidal behavior, which has opened up a new field of research in which relevant results are emerging. This could be of interest when designing new suicide prevention programs worldwide.

Given the potential usefulness of this research, future studies should aim to overcome the limitations of the current literature.

## Author contributions

ED-G and PF-B participated in the concept and writing of this manuscript. Both authors approved the final version of the manuscript.

### Conflict of interest statement

The authors declare that the research was conducted in the absence of any commercial or financial relationships that could be construed as a potential conflict of interest.
